# Stability and change in maternal wellbeing and illbeing from pregnancy to three years postpartum

**DOI:** 10.1007/s11136-024-03730-z

**Published:** 2024-07-11

**Authors:** Lilian Mayerhofer, Ragnhild Bang Nes, Baeksan Yu, Ziada Ayorech, Xiaoyu Lan, Eivind Ystrom, Espen Røysamb

**Affiliations:** 1 PROMENTA Research Center, Oslo University, Oslo, Norway; 2 Division of Mental and Physical Health, Norwegian Institute of Public Health, Oslo, Norway; 3 Department of Philosophy, Classics, and History of Arts and Ideas, University of Oslo, Oslo, Norway; 4 Gwangju National University of Education, Gwangju, South Korea; 5 PsychGen Centre for Genetic Epidemiology and Mental Health, Norwegian Institute of Public Health, Oslo, Norway

**Keywords:** Wellbeing, Illbeing, Polygenic indices, Motherhood, MoBa, Medical birth registry of norway

## Abstract

**Purpose:**

Motherhood affects women’s mental health, encompassing aspects of both wellbeing and illbeing. This study investigated stability and change in wellbeing (i.e., relationship satisfaction and positive affect) and illbeing (i.e., depressive and anxiety symptoms) from pregnancy to three years postpartum. We further investigated the mutual and dynamic relations between these constructs over time and the role of genetic propensities in their time-invariant stability.

**Data and methods:**

This four-wave longitudinal study included 83,124 women from the Norwegian Mother, Father, and Child Cohort Study (MoBa) linked to the Medical Birth Registry of Norway. Data were collected during pregnancy (30 weeks) and at 6, 18 and 36 months postpartum. Wellbeing and illbeing were based on the Relationship Satisfaction Scale, the Differential Emotions Scale and Hopkins Symptoms Checklist-8. Genetics were measured by the wellbeing spectrum polygenic index. Analyses were based on random intercept cross-lagged panel models using R.

**Results:**

All four outcomes showed high stability and were mutually interconnected over time, with abundant cross-lagged predictions. The period of greatest instability was from pregnancy to 6 months postpartum, followed by increasing stability. Prenatal relationship satisfaction played a crucial role in maternal mental health postpartum. Women’s genetic propensity to wellbeing contributed to time-invariant stability of all four constructs.

**Conclusion:**

Understanding the mutual relationship between different aspects of wellbeing and illbeing allows for identifying potential targets for health promotion interventions. Time-invariant stability was partially explained by genetics. Maternal wellbeing and illbeing develop in an interdependent way from pregnancy to 36 months postpartum.

**Supplementary Information:**

The online version contains supplementary material available at 10.1007/s11136-024-03730-z.

## Introduction

The birth of a child may substantially influence women’s mental health. A comprehensive understanding of mental health comprises both positive (i.e., wellbeing) and negative (i.e., illbeing) states. Wellbeing encompasses different aspects of one’s life. Positive affective experiences, like joy, are a part of subjective wellbeing [1], whereas relationship with others is a key aspect of both social and psychological wellbeing [2]. Illbeing often refers to depressive and anxiety symptoms. Wellbeing is not the diametral opposite of illbeing, but partly distinct entities [3, 4]. Thus, including both wellbeing and illbeing indicators provides a more realistic and holistic measure of maternal mental health.

Although romanticized by common sense [5], motherhood is often characterized by social isolation and feelings of “personal disconnection” and “insufficient support” [6, 7]. Thus, women’s relationship with their partner is a crucial source of emotional and social support [8]. Previous studies pointed that women’s relationship satisfaction (RelSat) commonly decreases over time, regardless of parental status [9]. Still, a steeper decline tends to follow the birth of a child [10]. This may partly reflect unfulfilled expectations regarding work division at home, with women in heterosexual couples often responsible for much more of the housework and childcare than they first expected [11, 12]. Another factor may be less quality time spent with their partners [13]. Despite this decline, prenatal RelSat remains highly important, predicting later postpartum life satisfaction and depressive symptoms in women [14, 15].

Another facet of wellbeing is positive affect (PosAff), referring to pleasant emotions such as happiness, and joy [16]. PosAff is generally associated with better mental and physical health [17] and particularly beneficial during pregnancy, protecting against harmful birth-related outcomes [18–20]. Despite the importance of PosAff, most previous studies focused exclusively on other wellbeing measures, leaving PosAff trajectories frequently unexplored. Overall, maternal wellbeing peaks during pregnancy /early postpartum (the “baby honeymoon”) and declines from 6 months postpartum [14, 21]. Multiple stress factors contribute to this decline, such as the return to work and restricting recreational and social activities [22].The perinatal period is a point of increased stress due to increasing burden of responsibility [6]. Approximately 13% of pregnant women fulfill the criteria for a major depressive disorder [23]; and up to 25% experience anxiety symptoms [24]. From 6.3 to 9.5% of pregnant women experience both types of symptoms [25]. Maternal illbeing during pregnancy predicts countless harmful outcomes to the mother and her offspring [26]. Prenatal depressive symptomsincreased the risk of postpartum depression and adverse birth outcomes [27]. Prenatal anxiety symptoms were linked to lower maternal quality of life and offspring behavioral problems [28]. Anxiety symptoms reportedly decrease immediately after birth but increase again after 6 weeks [29].

Both wellbeing and illbeing are influenced by environmental and genetic factors [30]. The multifaceted nature of poverty diminishes social wellbeing and increases negative affect by reducing social interactions [31]. Poverty also increases the risk of experiencing depressive and anxiety symptoms up to 3 times, and these symptoms might hinder education and skills acquisition [31, 32]. As for genetics, the average heritability of wellbeing has been estimated to 40% in a previous metanalysis [33], whereas the heritability of illbeing is 30 to 40% [34, 35]. Single nucleotide polymorphisms (SNPs) are variants that influence complex traits and capture individual specific genetic propensity [36]. SNPs are identified through Genome-Wide Association Studies (GWAS) and are the basis for polygenic indices (PGIs) [37]. Different PGIs have consistently predicted maternal mental health [38], but are mostly limited to PGIs associated with illbeing traits.

**Fig. 1:**
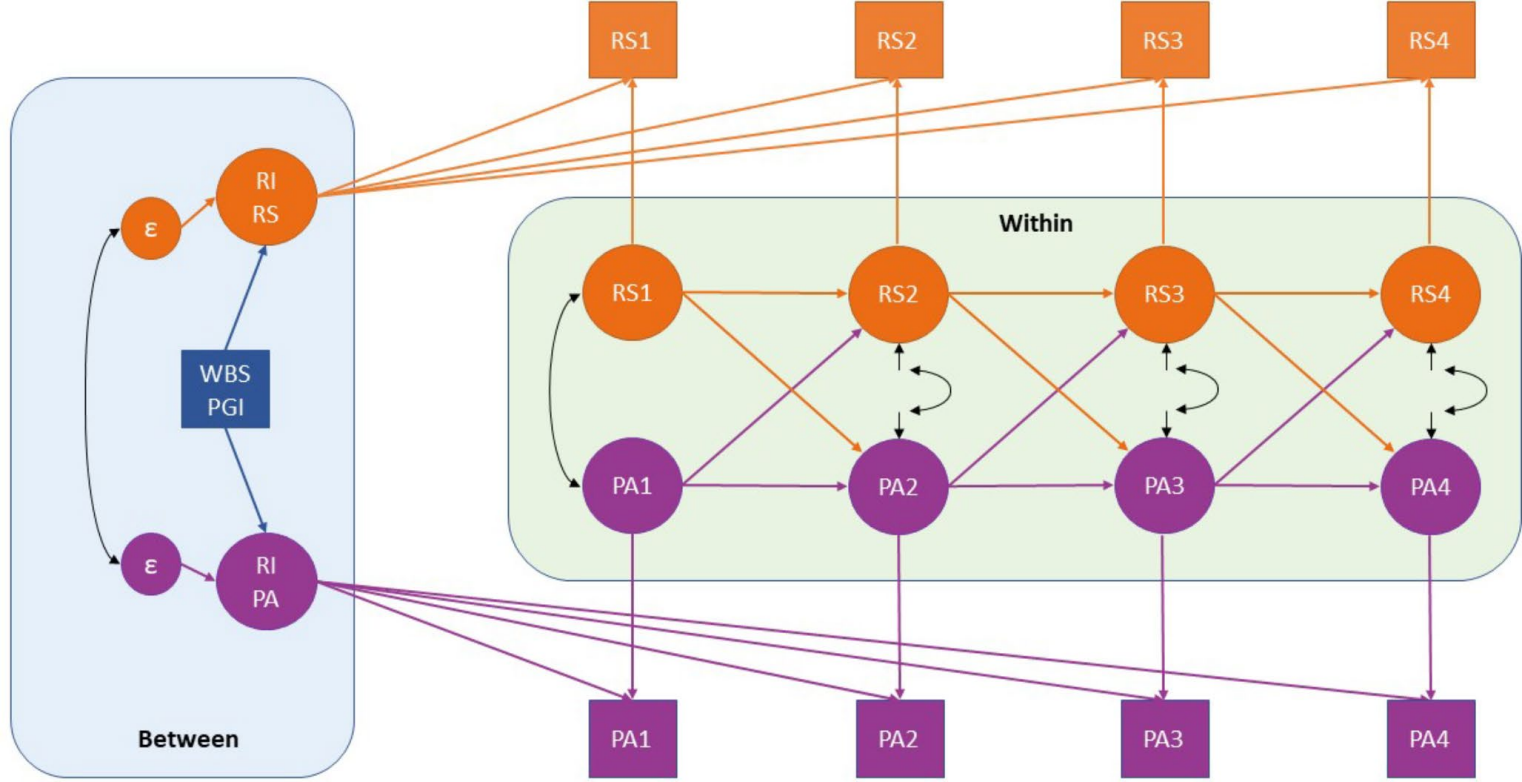
Theoretical Random Intercept Cross-Lagged Panel Model with Two Variables. WBS PGI: Wellbeing spectrum polygenic index; RI: Random Intercept; RS: Relationship Satisfaction; PA: Positive Affect. Numbers indicate the different timepoints (1 to 4). “Between” indicates the “between-person” processes (also referred as “time-invariant stability”), whereas “within” indicates “within-person” processes, composed of stability (auto-regressive paths) and change (cross-lagged paths)

Most previous studies have used conventional cross-lagged panel models (CLPMs) to investigate transitions in mental health [39–41]. These models examine stability over time through autoregressive paths and change through reciprocal relationships between different constructs (cross-lagged paths) [42]. However, conventional CLPMs assumes that any stability over time is only due to past levels, ignoring the possibility that some traits remain consistent regardless of what happens in the short term [42]. Establishing a latent random intercept (RI) could account for the time-invariant stability, explaining between-person variability [42]. The subsequent auto-regressive and cross-lagged paths represent within-person processes over time. Different constructs can also co-develop over time, as indicated by the residual correlations. A theoretical model for the RI-CLPM is shown in Fig. 1.

The current study followed a large sample of women from pregnancy to 3 years postpartum (total *N* = 80,000). The extensive sample size and follow-up period allowed for a comprehensive investigation of the dynamics between wellbeing and illbeing, in terms of maternal relationship satisfaction, positive affect, depressive and anxiety symptoms. We adopted a RI-CLPM framework to better account for the time-invariant stability and expanded the traditional RI-CLPM to include four constructs, as previous studies often included two variables only. Additionally, we examined the importance of mothers’ genetic propensity (i.e., PGI) to the wellbeing spectrum (WBS) to time-invariant stability (i.e., RIs) in wellbeing and illbeing [43]. Thus, our aims were to investigate (a) the importance of women’s genetic propensity to wellbeing (i.e., WBS PGI) in predicting time-invariant stability in maternal RelSat, PosAff, and depressive and anxiety symptoms and (b) the complex relationship between RelSat, PosAff, and depressive and anxiety symptoms from late pregnancy to 3 years postpartum. The complete theoretical model is shown in Figure S1. We hypothesized that (a) WBS PGI can predict the stability of all four constructs, and (b) there are mutual relations between all four constructs in their change over time.

## Methods

### Participants and procedures

Data were drawn from the Norwegian Mother, Father and Child Cohort Study (MoBa) [44] and the Medical Birth Registry (MBRN), which contains information about all births in Norway [45]. MoBa is a population-based pregnancy study conducted by the Norwegian Institute of Public Health. Participants were recruited from all over Norway from 1999 to 2008. The current study is based on version 12 of the quality-assured data files released for research in 2020. The establishment of MoBa and initial data collection were based on a license from the Norwegian Data Protection Agency and approval from The Regional Committees for Medical and Health Research Ethics. The MoBa cohort is currently regulated by the Norwegian Health Registry Act. The current study was approved by The Regional Committees for Medical and Health Research Ethics (reference number 318,756).

In total, 95,136 women were included in MoBa. For this study, we included only the first pregnancy after MoBa recruitment. If the pregnancy resulted in twins, mothers still submitted only one mental health self-report. Next, we excluded 324 women whose pregnancy resulted in stillbirth. Then 11,688 mothers were excluded for not taking part at any of the four timepoints included in this study. The final sample thus comprised 83,124 women.

This four-wave panel study included measurements from late pregnancy (T1, at 30 weeks) and then at 6 (T2), 18 (T3) and 36 (T4) months after birth. Participation varied across the four waves. At T1, 79,398 (95.51%) women responded, decreasing to 74,618 (89.77%) at T2, 63,919 (76.9%) at T3 and 48,756 (58.65%) at T4. Despite the selective attrition, previous studies indicated that the resulting bias in MoBa mainly affected means and prevalence, and not estimates of associations between variables, which is the focus of the current study [46, 47].

### Measurements

**Relationship satisfaction** was measured by the 5-item (T4) and the 10-item (T1, T2, T3) Relationship Satisfaction Scale (RSS) [48]. Questions are, for example, “I am very happy with our relationship” and “We agree on how our child should be raised”. Responses included 6 options from “agree completely” to “disagree completely”. The mean composite score was calculated for each wave. Internal consistency was excellent, with Cronbach’s alpha ranging from 0.90 to 0.93.

**Positive Affect** was measured by the Enjoyment subscale from the Differential Emotional Scale (DES) [49]. This 3-item subscale has 5 response options from “rarely or never” to “very often”. Examples of questions are “Feel glad about something” and “Feel happy”. The mean composite score was calculated for each wave. Internal consistency was good, with Cronbach’s alpha ranging from 0.81 to 0.83.

**Depressive and anxiety symptoms** were assessed through the Hopkins Symptoms Checklist-8 (SCL-8) [50–52]. This scale comprises 4 items each for depressive and anxiety symptomsand 4 response options, from “not bothered” to “very bothered”. One example of depressive symptom is “Feeling blue”, and of anxiety symptom is “Nervousness or shakiness inside”. The mean composite scores for depressive and anxiety symptoms were calculated separately. Both variables had good internal consistency, with Cronbach’s alphas ranging from 0.73 to 0.77.

**Genetic influence** was measured by the WBS PGI [43] and genetic information was available from 68,227 (82.1%) women. We used beta weights from the WBS GWAS. The WBS PGI was calculated using LDPred, a Bayesian approach that uses a prior on the expected polygenicity of a trait (assumed fraction of non-zero effect markers) and adjusts for linkage disequilibrium (LD) based on a reference panel to compute SNPs weights [53]. Genotypes were coordinated with the summary statistics, leaving 800,700 SNPs for the WBS GWAS. LD adjustment was performed using the European subsample of the 1000 Genomes genotype data as LD reference panel. The weights were estimated based on the heritability explained by the markers in the GWAS summary statistics and the assumed fraction of markers with non-zero effects. The LDpred PGI was created with the “–score” command in plink2. A full description of the genotype quality control is available at the supplementary material.

In all variables, a higher score indicates a greater presence of the respective construct. For instance, a higher score in RelSat means that women are more content in their relationships. A higher WBS PGI indicates a higher genetic propensity to the wellbeing spectrum, defined as higher wellbeing (e.g., PosAff) and lower illbeing (e.g., depressive symptoms) [43]. Variables were log transformed to reduce distribution’s skewness.

### Statistical analyses

Analyses were run in R using the package “Lavaan” [54]. Analyses were based on robust maximum likelihood estimation (MLR), where missing data were handled through full information maximum likelihood (FIML). Analyses were based on RI-CLPM [42]. Coefficient significance threshold was established as *p* < 0.01, based on the large sample size.

First, we used the WBS PGI to predict the time-invariant stability (latent RIs) of maternal RelSat, PosAff, depressive and anxiety symptoms. Then we analyzed (latent) within-person processes over time. We started by constraining all autoregressive and cross-lagged paths (model 1) [55, 56]. Next, we removed the constraints between T1 and T2 (model 2). Finally, model 3 is a fully unconstrained model. We then compared the fit among these models through chi-square tests and fit indices [57], such as the Comparative Fit Index (CFI), Tucker‒Lewis Index (TLI), Akaike Information Criteria (AIC), Bayesian Information Criteria (BIC), Conditional Log-likelihood (LogLik) and Root Mean Square Error of Approximation (RMSEA) [58]. Criteria for CFI and TLI was established as ≥ 0.95 and RMSEA as ≤ 0.08 [59, 60].

## Results

### Descriptive statistics

**Fig. 2:**
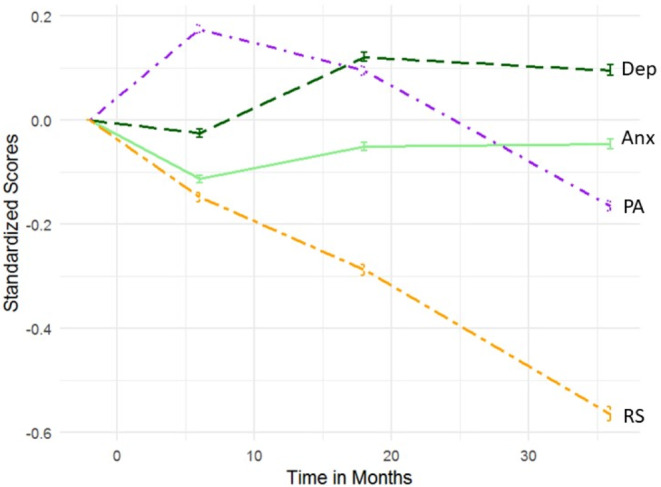
Standardized Averages and Trajectories. Standardized scores with 95% confidence intervals. Time counted in months, where 0 represents time of delivery. RS: Relationship Satisfaction; PA: Positive Affect; Dep: Depressive symptoms; Anx: Anxiety Symptoms

**Fig. 3:**
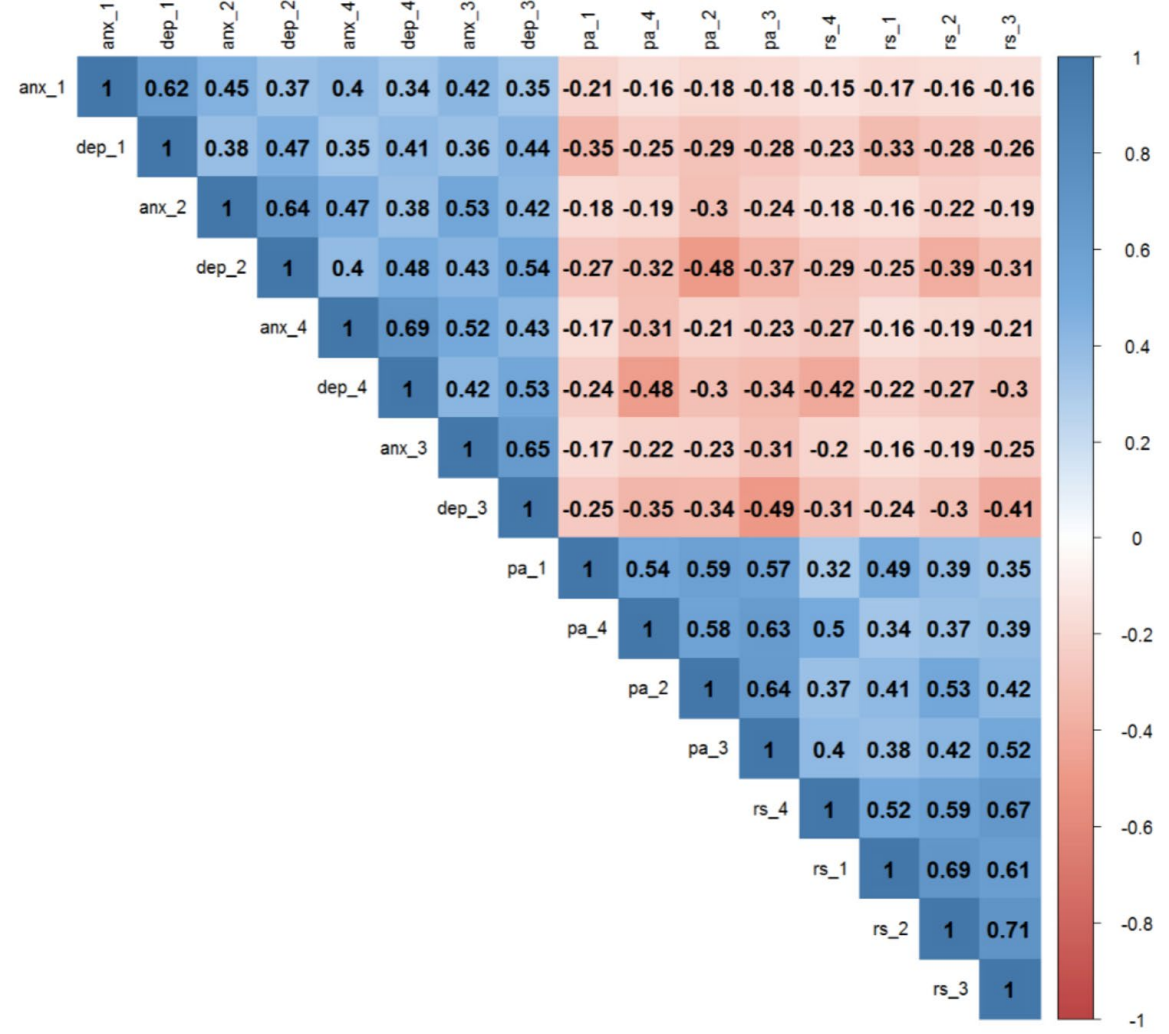
Correlation Plot. RS: Relationship Satisfaction; PA: Positive Affect; Dep: Depressive symptoms; Anx: Anxiety symptoms. Numbers indicate the different timepoints (1 to 4)

Descriptive statistics for RelSat, PosAff, depressive and anxiety symptoms are shown in Table 1. The mean RelSat score ranged from 5.01 to 5.36; PosAff, from 3.78 to 3.99; depressive symptoms, from 1.31 to 1.37; and anxiety symptoms, from 1.18 to 1.23. The trajectories of all four variables over time standardized on T1 scores are shown in Fig. 2. Correlations between all observed measures ranged between − 0.15 and 0.69 and are presented in Fig. 3. Partial correlations controlling for age, education and income are available at the supplementary material (Fig. S2). The intra-class correlations for RelSat, PositAff, anxiety and depressive symptoms were 0.63, 0.58, 0.49 and 0.49, respectively.

### Model comparison and fit

Overall, model 3 (all paths freely estimated) had the best performance based on fit indices (Table 2), with CFI = 0.997, TLI = 0.993 and RMSEA = 0.019. Model 3 was also the best model based on chi-square tests (model 3 against model 1 had Δχ^2^(32) = 1823 (*p* < 0.001), and against model 2 had Δχ^2^(16) = 289, *p* < 0.001). Model 3 showed good fit according to established model fit criteria. In model 3, we have also tested constraining the grand means for the four constructs, but these constrains were untenable (Δχ2(12) = 20,831, *p* < 0.0001).

### Model composition

#### Stability

**Fig. 4:**
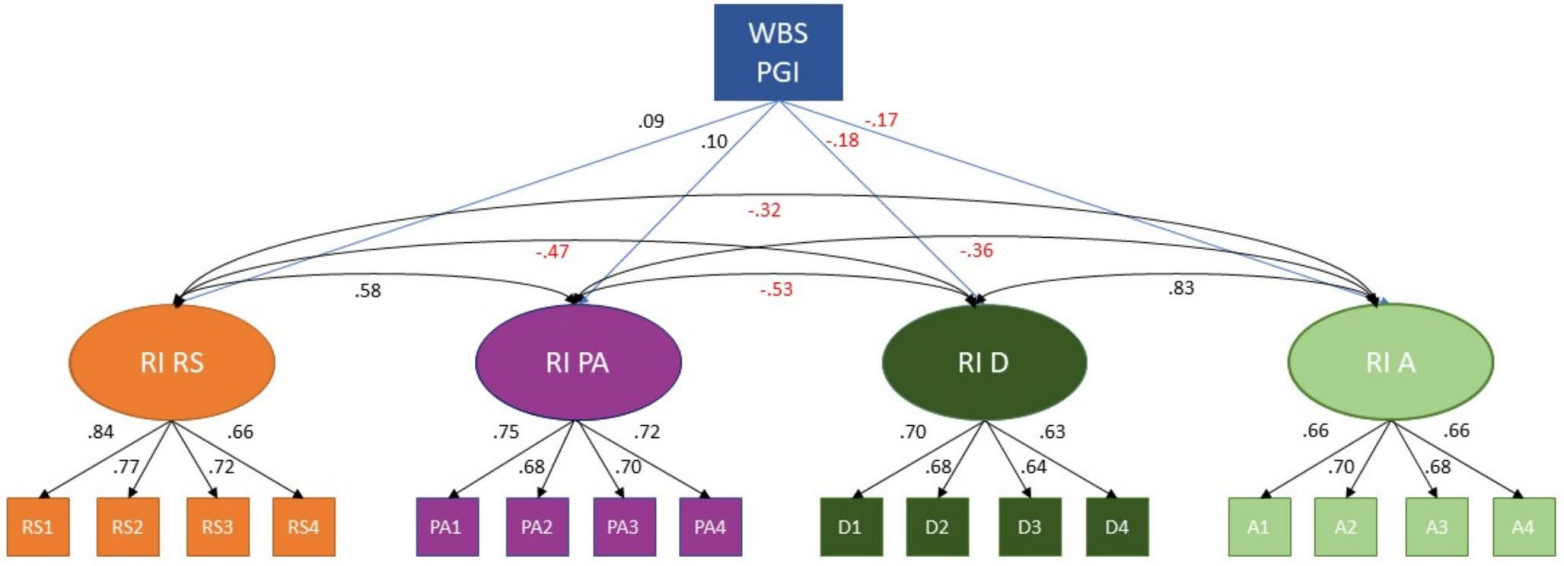
Time-invariant Stability of the Constructs (Between-Person Processes). WBS PGI: Wellbeing spectrum polygenic index; RI: Random Intercept; RS: Relationship Satisfaction; PA: Positive Affect; D: Depressive; A: Anxiety. Numbers indicate the different timepoints (1 to 4). All coefficients are standardized and have *p* < 0.001

All four variables showed a high level of stability, with RI loadings to observed measures ranging from 0.60 to 0.84 (standardized). RIs explained 36–70% of the variability in the observed measurements. Correlations between the four RIs ranged from − 0.32 to 0.83. Following our hypothesis 1, according to the guidelines followed by Orth et al., we found that WBS PGI had a medium effect positively predicting the RIs for RelSat and PosAff (*β =* 0.09 and 0.10, respectively) and large effect negatively predicting depressive and anxiety symptoms (*β* = − 0.18 and − 0.17, respectively) [61]. The theoretical model and standardized coefficients are presented in Fig. 4. Although we have focused on WBS PGI loading to RIs in this study, we have also tested WBS loading to observed measures in the different timepoints. These results are available in Table S1 (supplementary). Overall, WBS explained much more of the time-invariant variance than the average observed measures (0.81% against 0.66% for RS; 1.00% against 0.76% for PA; 3.24% against 1.69% for depressive symptoms and 2.89% against 1.46% for anxiety symptoms).

**Fig. 5:**
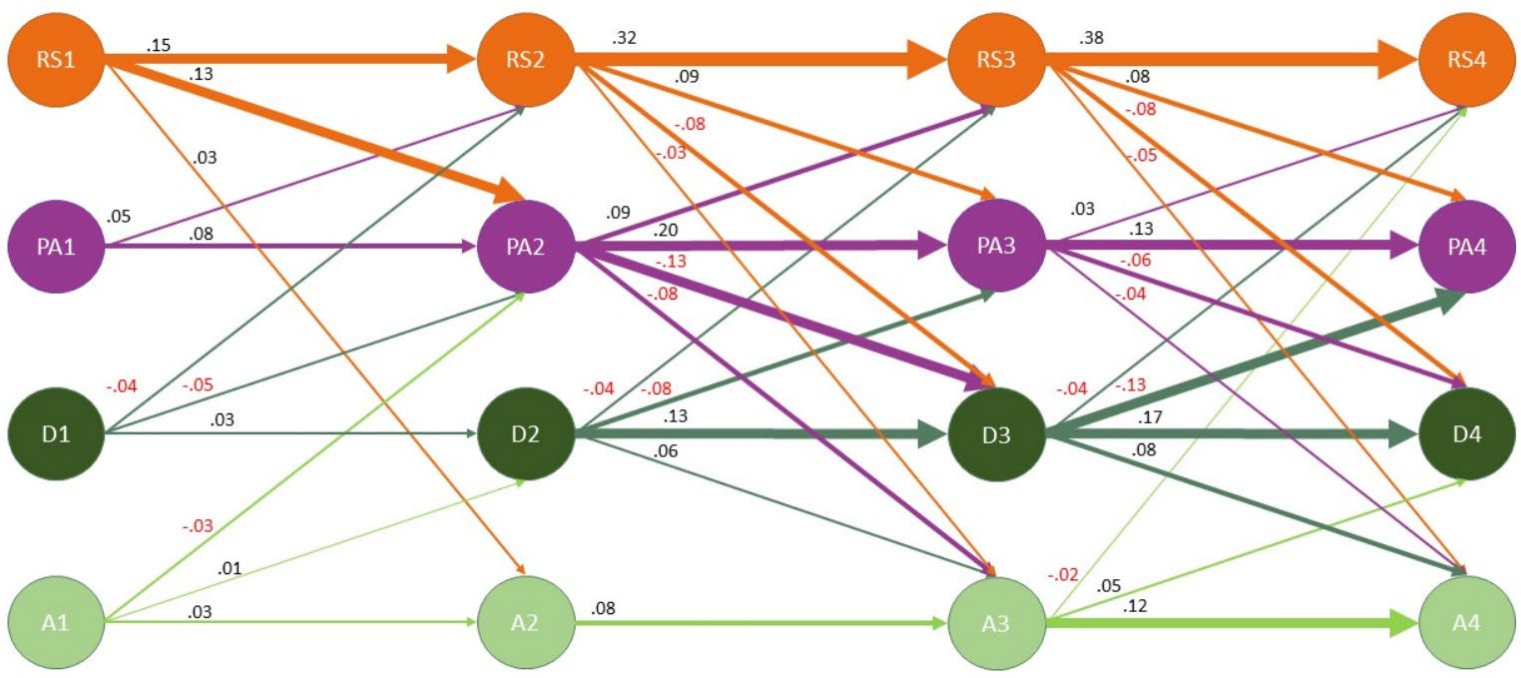
Stability and Change in the Constructs (Within-Person Processes). All coefficients shown are standardized and significant (*p* < 0.001). Nonsignificant coefficients were excluded from the figure to improve readability. The width of the arrows is proportional to the coefficient size. RS = Relationship satisfaction; PA = Positive affect; D = Depressive; A = Anxiety. Numbers represent the different timepoints (1 to 4)

Autoregressive coefficients increased for all measures postpartum compared to the pre-to-postpartum transition, with the highest values for RelSat especially between 6 and 36 months (*β* from 0.32 to 0.38). Once controlling for the time-invariant stability (RI), prenatal depressive symptoms had a small effect predicting depressive symptoms 6 months postpartum (*β* = 0.03, *p <* 0.001). All standardized auto-regressive coefficients are presented in Fig. 5.

#### Change

Following our hypothesis 2, we found abundant mutual relationships between all four constructs. All standardized coefficients are also presented in Fig. 5.Prenatal RelSat had a small effect positively predicting anxiety symptoms at 6 months postpartum (*β =* 0.03) with this prediction becoming negative at later timepoints. PosAff and depressive symptoms were closely related in the postpartum period. Prenatal depressive symptoms did not predict anxiety symptoms postpartum, butpredicted in later timepoints. Overall, anxiety symptoms developed more independently, either with a small or no effect predicting the other constructs (*β* from − 0.03 to 0.05).

**Fig. 6:**
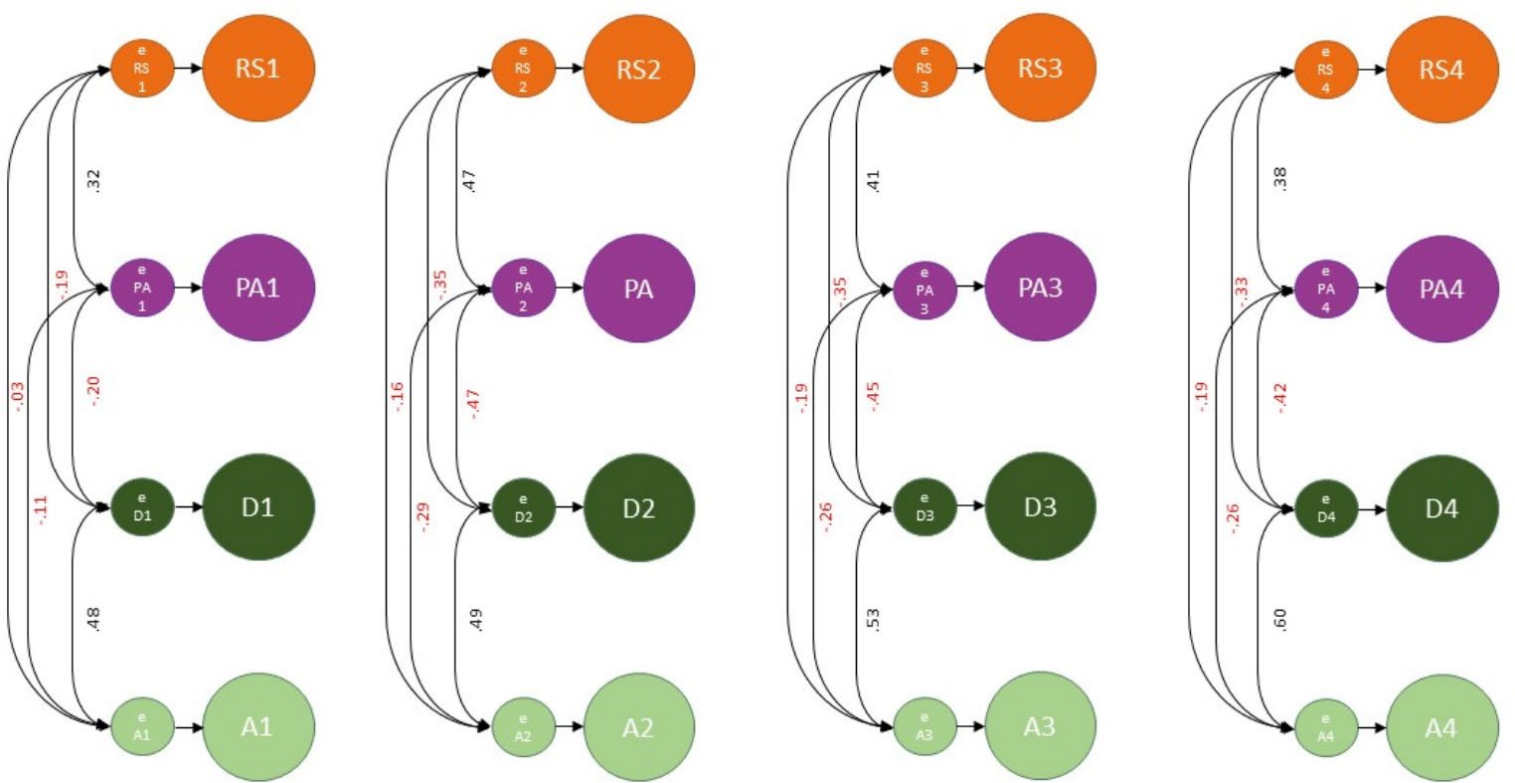
Time-Specific Residual Correlations. All coefficients were standardized and significant. RS = Relationship satisfaction; PA = Positive affect; D = Depressive; A = Anxiety. Numbers represent the different timepoints (1 to 4)

Time specific residual correlations refer to the co-development or co-change between different constructs [62]. These correlations ranged from − 0.47 to 0.60. Correlations between RelSat and PosAff, as well as between depressive and anxiety symptoms, were positive. Correlations between wellbeing and illbeing constructs were negative. These coefficients are shown in Fig. 6.

## Discussion

This study addressed the stability and change in maternal RelSat, PosAff, and depressive and anxiety symptoms from late pregnancy to 3 years postpartum. We investigated (i) the importance of women’s genetic propensity to wellbeing using the WBS PGI to the time-invariant stability in all four constructs and (ii) the mutual relationships between the constructs over time. The WBS PGI predicted all RIs, and all four key constructs showed interconnected paths over time. These findings reinforce concepts from the family systems theory [63], where family is regarded as an emotional unit and there are complex interactions within this unit (e.g., the quality of the relationship of the parents affecting women’s mental health). Here we discuss our findings considering prior work, the general implications of our findings, and the limitations of this study.

### Genetic influences

The WBS PGI represents a part of women’s genetic propensity to experience higher wellbeing and lower illbeing. WBS PGI predicted time-invariant stability of RelSat, PosAff, depressive and anxiety symptoms. This means that genetics contributed to differences between women in all four constructs. Predictions were greater for illbeing than for wellbeing, with moderate effects to the former and large to the latter. This could be due to the nature of the WBS PGI, including not only life satisfaction and positive affect, but also neuroticism and depressive symptoms [43]. The variance explained by the WBS PGI (from 0.81 to 3.24%) was virtually the double of what was reported previously [64].

PGI effects in general are reported to be small [65]. One reason is that PGIs capture only a fraction of SNP-heritability, leading to a gap between the variance captured by PGIs and heritability estimates [66]. Still, the stability of wellbeing and illbeing has been reported to be highly heritable [67, 68]. Our study is unique in presenting the effect of the WBS PGI in the time-invariant stability, pointing to the same direction of high genetic influence in the stability of traits that are shown by twin studies.

### Stability

Agreeing with previous literature, RelSat, PosAff, depressive and anxiety symptoms presented a moderate to high level of stability [69]. Although the correlation between RIs for depressive and anxiety symptoms was high (0.83), their correlations with the other constructs differed. Overall, the correlations for depressive symptoms were greater than the respective correlations for anxiety symptoms. This may indicate that depressive and anxiety symptoms are not as similar as previously described [70, 71] and might respond differently to a same predictor [72], especially given their association with RelSat and PosAff [73].

From late pregnancy to 6 months postpartum great instability occurs [14, 74]. From 6 to 36 months, the autoregressive coefficients increased for all four constructs. Contradicting a previous finding [75], we found that prenatal depressive symptoms have a small effect predicting postnatal depressive symptoms. This is at odds with one previous finding [76], but most former studies have not distinguished time-specific symptoms from trait-like symptoms. Depressive symptoms in the postpartum can arise due to situation-specific stress, as the stress associated with child care [77].

### Change

#### Pre to postpartum

Prenatal RelSat positively predicted anxiety symptoms postpartum. In other words, when all other factors are held constant, mothers with higher RelSat during pregnancy tend to have greater levels of anxiety symptoms postpartum. This finding is in line with previous reports [78] and may reflect unfulfilled expectations, for example with regards to the division of work with one’s partners [79] or less quality time spent with one’s partners [13].

Prenatal anxiety symptoms positively predicted depressive symptoms postpartum, but the effect was smaller than the auto-regressive path for depressive symptoms in the same period. This finding contradicts previous research, where a prenatal history of anxiety symptoms was said to be a greater risk factor for postnatal depressive symptoms than a history of depressive symptoms itself [80, 81]. One possible explanation for why anxiety symptoms predicted depressive symptoms is that the former usually involve negative thinking patterns and excessive worry. Once these symptoms are carried over the postpartum period, they increase the risk of developing depressive symptoms. Contradicting previous studies, prenatal depressive symptoms did not predict postpartum anxiety symptoms [82]. In other words, although time-invariant stability for depressive and anxiety symptoms are strongly positively correlated, depressive symptoms specifically observed during pregnancy did not influence anxiety symptoms following birth.

#### Postpartum

RelSat consistently predicted PosAff, depressive and anxiety symptoms at all timepoints. Yet, depressive and anxiety symptoms hardly predicted RelSat from 6 to 36 months. These findings contradict previous findings [83] and highlight that RelSat levels remained robust despite women experiencing depressive and anxiety symptoms. This “damper” effect could point to women over-performing in family situations due to traditional gender roles and expectations.

In accordance with the previous literature, PosAff and depressive symptoms were closely connected postpartum [84]. PosAff reduced future levels of depressive symptoms and increased RelSat. Possible explanations include that PosAff could help women reframe negative thoughts and adopt a more balanced perspective. PosAff also encourages engagement in social and pleasurable activities, improving the quality of the relationship. As described by the broaden-and-build theory, PosAff broaden one’s momentary thought-action repertoire (e.g., “contentment” could spark the urge to savour and integrate with others), building social and psychological resources [85]. Interestingly, while RelSat, PosAff and depressive symptoms are closely interconnected in the postpartum, anxiety symptoms seem to “live a life of their own”. RelSat, PosAff, and depressive symptoms modestly predict anxiety symptoms, while the latter had minimal or no impact on the other three constructs over all time points. This could be due to the distinct nature of anxiety, focusing more on specific fears or worries, which might not have as broad an impact on overall relationship quality or PosAff.

#### Co-development

Residual correlations were overall high, and represent changes that occur jointly, as a tango for variables. For example, when relationship satisfaction increased from one time point to the next, this change did not occur in isolation, but rather in conjunction with increases in positive affect and reductions in anxiety and depression. These co-developments may suggest the presence of common unobserved factors that influence two or more constructs simultaneously, for example positive or negative life events, or other risk and protective factors with multifinal effects. Co-development may also be due to concurrent or short-term causal effects between the variables, for example increased relationship satisfaction causing reduced levels of depression. While the exact nature of the causal mechanisms involved cannot the delineated in the current design, the finding of substantial co-development suggests a potential for health promotive interventions to have important effects across different conditions.

### Implications

Investigating stability and change in RelSat, PosAff, depressive and anxiety symptoms contribute to a better understanding of the nature of these constructs and guiding evidence-based-health-promotion-measures. The inclusion of genetically sensitive designs gets us closer to the directional impact the stability of the four constructs, given that environmental factors cannot change DNA.

Our study highlights the important role of RelSat protecting against both depressive and anxiety symptoms in mothers. Considering this protective role and the sustained decline in RelSat, measures that promote RelSat should be encouraged. PosAff and depressive symptoms had a strong, mutual relationship after birth. Therefore, measures aiming at preventing depressive symptoms postpartum could include those that promote PA. Co-development can also be important for intervention strategies. If constructs consistently co-develop in a positive manner, such as RelSat and PosAff, interventions targeting one variable might have spillover effects on the other.

Our findings also point to important mental health screening strategies for women. There are limited associations between pre and postpartum measures, and these two periods seem to develop somewhat independently. For example, depressive symptoms in the prenatal period have a small effect predicting these same symptoms in the postpartum. Therefore, mental health screening for women should be recommended in both pre and postpartum periods. A recent systematic review pointed to the same direction, in which perinatal screening for depression and anxiety symptoms compared with no screening improved maternal mental health [86]. In Norway, national guidelines recommend that health services identify women with perinatal mental conditions, but systematic screening is not nationally endorsed [87]. New factors influencing wellbeing and illbeing come into play after birth, and further studies investigating these factors are encouraged.

### Strengths and limitations

This study included a large sample and substantial follow-up period. Nonetheless, limitations exist and should be addressed. Participation rate among pregnancies invited to MoBa was 41% [44]. As participants are mainly educated and Western (Norwegian women), our findings may not extrapolate to other cohorts and ancestries. Observational studies are also inherently subject to time-varying confounders. Wellbeing is a broad concept, encompassing constructs beyond those included in this study. We did not focus on the role of potential important covariates, such as education, income, and age. Still, the potential confounding brough by these variables was very limited (see Fig S2).

## Conclusion

Giving birth and raising a new member of the family can be challenging to women’s mental health. RelSat, PosAff, depressive and anxiety symptoms are highly stable over time. Still, the challenges associated with motherhood lead to changes in these constructs, building complex and mutual relationships between them. Adopting an analytical strategy that accounts for stability and change contributes to better understanding the dynamic transitions and mutual relations between RelSat, PosAff, depressive and anxiety symptoms. The period of greater instability was from pre to postpartum, followed by increasing stability. The stable, trait-like component of RelSat, PosAff, depressive and anxiety symptoms can be partially explained by women’s propensity to wellbeing, revealing once more the dynamic relationship between wellbeing and illbeing. Future studies are encouraged to expand to other facets of wellbeing and illbeing, such as flourishing or loneliness; and to consider the time-invariant stability when addressing stability and change.

**Table 1:** Mean scores

	T1		T2		T3		T4	
	mean	SD	mean	SD	mean	SD	mean	SD
Relationship satisfaction	5.36	0.63	5.27	0.72	5.18	0.78	5.01	0.90
Positive affect	3.88	0.63	3.99	0.68	3.94	0.67	3.78	0.67
Depressive symptoms	1.32	0.42	1.31	0.44	1.37	0.47	1.36	0.49
Anxiety symptoms	1.23	0.37	1.18	0.35	1.21	0.36	1.21	0.37

SD = standard deviation; T1 = timepoint 1 (30 weeks of pregnancy); T2 = timepoint 2 (6 months after birth); T3 = timepoint 3 (18 months after birth); T4 = timepoint 4 (36 months after birth)

**Table 2:** Model Fit Comparison among the different models tested

Model	CFI	TLI	AIC	BIC	LogLik	RMSEA
1	0.994	0.990	867797.067	868599.093	-433812.534	0.022
2	0.997	0.993	866270.147	867221.387	-433033.073	0.018
3	0.997	0.993	866010.714	867111.168	-432887.357	0.019

Model 1: all within-level paths constrained over time; Model 2: transition between waves 1 and 2 (pre-to-postbirth) unconstrained, whereas postbirth remained constrained; Model 3: all within-level paths unconstrained over time. CFI = Comparative Fit Index; TLI = Tucker–Lewis Index; AIC = Akaike Information Criteria; BIC = Bayesian Information Criteria; LogLik = Conditional Log-likelihood; RMSEA = Root Mean Square Error of Approximation

## Electronic supplementary material

Below is the link to the electronic supplementary material.

Supplementary Material 1

## Data Availability

Data from the Norwegian Mother, Father and Child Cohort Study and the Medical Birth Registry of Norway used in this study are managed by the national health register holders in Norway (Norwegian Institute of public health) and can be made available to researchers, provided approval from the Regional Committees for Medical and Health Research Ethics (REC), compliance with the EU General Data Protection Regulation (GDPR) and approval from the data owners. The consent given by the participants does not open for storage of data on an individual level in repositories or journals. Researchers who want access to data sets for replication should apply through helsedata.no. Access to data sets requires approval from The Regional Committee for Medical and Health Research Ethics in Norway and an agreement with MoBa. All R codes used are available from the first author upon request.
