# Supplementary material for: Stability and change in maternal wellbeing and illbeing from pregnancy to three years postpartum
Source: Qual Life Res. 2024 Jul 11;33(10):2797–808. doi: 10.1007/s11136-024-03730-z (PMC11452533; doi:10.1007/s11136-024-03730-z)
Supplement: Supplementary file 1 — Supplementary Material 1 [file 11136_2024_3730_MOESM1_ESM.docx]

**Figure S1**


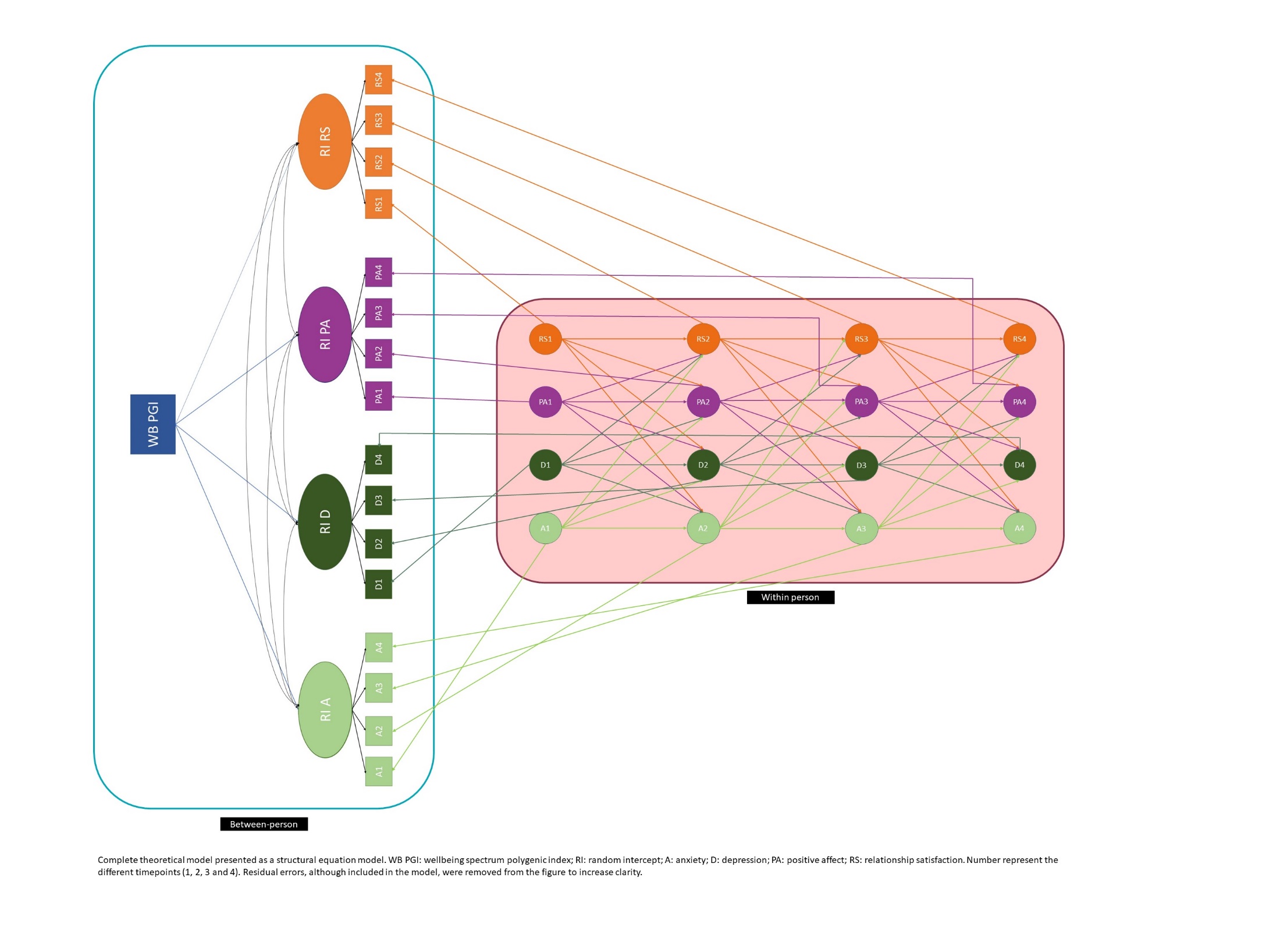
*Complete Theoretical Model*

Complete theoretical model presented as a structural equation model. WB PGI: wellbeing spectrum polygenic index; RI: random intercept; A: anxiety; D: depression; PA: positive affect; RS: relationship satisfaction. Number represent the different timepoints (1, 2, 3 and 4). Residual errors, although included in the model, were removed from the figure to increase clarity.

**Figure S2**


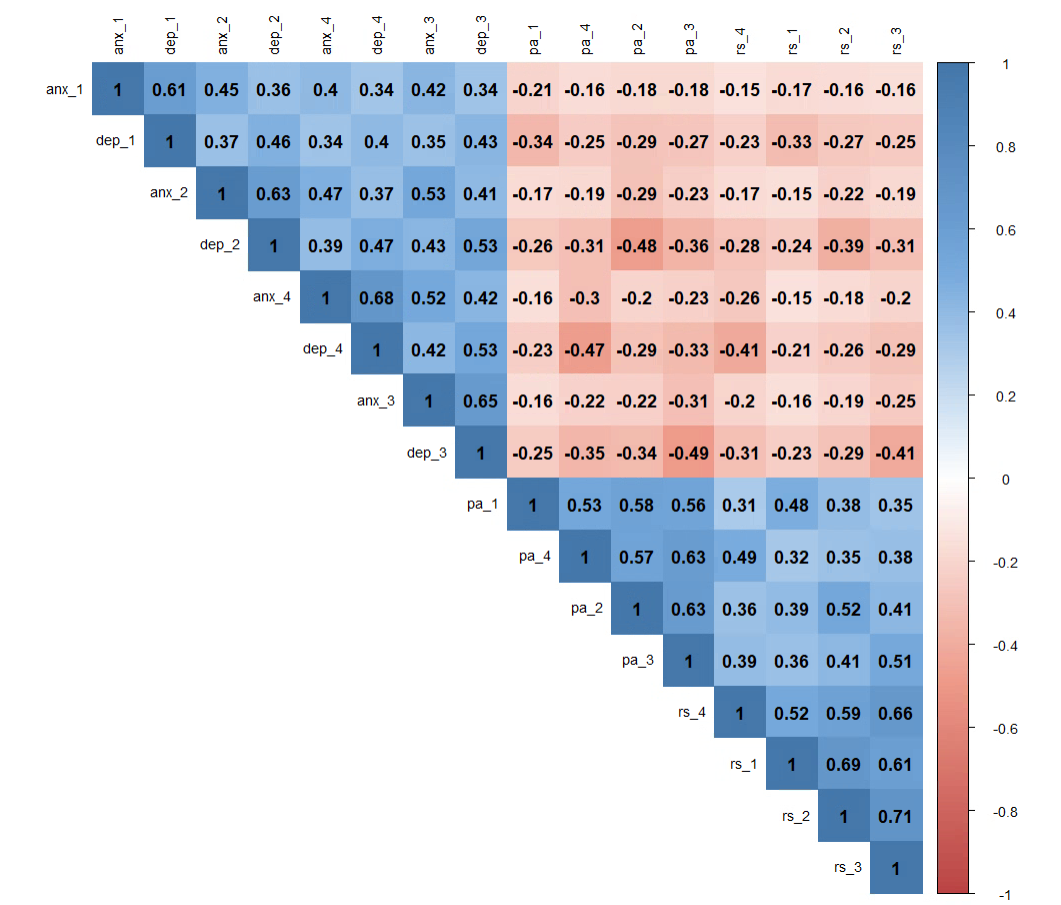
*Partial Correlations*

Partial correlations after controlling for income, educational level and age. Anx: anxiety; Dep: depression; PA: positive affect; RS: relationship satisfaction. Number represent the different timepoints (1, 2, 3 and 4).

Table S1

*Wellbeing Spectrum Polygenic Index Loading to Individual Measures*

|  | T1 | T2 | T3 | T4 |
| --- | --- | --- | --- | --- |
| Relationship Satisfaction | .064 | .082 | .084 | .095 |
| Positive  Affect | .068 | .088 | .095 | .098 |
| Depressive Symptoms | -.116 | -.129 | -.131 | -.144 |
| Anxiety  Symptoms | -.115 | -.117 | -.117 | -.134 |

All coefficients are standardized. T1 = timepoint 1 (30 weeks of pregnancy); T2 = timepoint 2 (6 months after birth); T3 = timepoint 3 (18 months after birth); T4 = timepoint 4 (36 months after birth).

Genotype Quality Control

Pre-imputation quality control (QC), phasing, imputation, and post-imputation QC have been conducted for MoBa data using the MoBaPsychGen pipeline, which was designed to handle genotyping data from large population-based samples of individuals with complex interrelatedness [1]. Phasing and imputation were performed with IMPUTE4.1.2_r300.3, using the publicly available Haplotype Reference Consortium release 1.1 panel as a reference. In total, 207,569 unique individuals (90% of the unique individuals included in the original MoBa study) and 6,981,748 SNPs passed the MoBaPsychGen pipeline. To identify a sub-population of European-associated ancestry, principal component analysis (PCA) was performed with 1000 Genomes phase 1 after LD-pruning (see Corefield et al., 2022 for full details). All subsequent genomic analyses included 20 principal components (PCs) to control for population stratification and genotyping batch to adjust for any batch effects. '

Reference

1. Corfield EC, Frei O, Shadrin AA, Rahman Z, Lin A, Athanasiu L, Akdeniz BC, Hannigan L, Wootton RE, Austerberry C, Hughes A, Tesli M, Westlye LT, Stefánsson H, Stefánsson K, Njølstad PR, Magnus P, Davies NM, Appadurai V, et al. The Norwegian Mother, Father, and Child cohort study (MoBa) genotyping data resource: MoBaPsychGen pipeline v.1. bioRxiv. 2022:2022.06.23.496289.

Regional Considerations:

The MoBa cohort includes women from different Norwegian regions. In the current study, we tested including geodata at the municipality level (n = 437 municipalities), accounting for the municipality where women were living by the occasion of the first timepoint in this study. Our analyses showed that municipality only accounted for less than 1% of the variance in the observed values of relationship satisfaction (η^2^ = 0.010), positive affect (η^2^ = 0.009), depressive symptoms (η^2^ = 0.007) and anxiety symptoms (η^2^ = 0.006).
